# Development and validation of assessment instrument for the perception and attitude toward tuberculosis among the general population in Indonesia: a Rasch analysis of psychometric properties

**DOI:** 10.3389/fpubh.2023.1143120

**Published:** 2023-09-28

**Authors:** Dian Ayu Eka Pitaloka, Ikhwan Yuda Kusuma, Hening Pratiwi, Ivan Surya Pradipta

**Affiliations:** ^1^Department of Pharmacology and Clinical Pharmacy, Faculty of Pharmacy, Universitas Padjadjaran, Sumedang, Indonesia; ^2^Center of Excellence in Higher Education for Pharmaceutical Care Innovation, Universitas Padjadjaran, Sumedang, Indonesia; ^3^Pharmacy Study Program, Faculty of Health, Universitas Harapan Bangsa, Purwokerto, Indonesia; ^4^Institute of Clinical Pharmacy, University of Szeged, Szeged, Hungary; ^5^Department of Pharmacy, Faculty of Health Sciences, Universitas Jenderal Soedirman, Purwokerto, Indonesia; ^6^Drug Utilization and Pharmacoepidemiology Research Group, Center of Excellence in Higher Education for Pharmaceutical Care Innovation, Universitas Padjadjaran, Sumedang, Indonesia

**Keywords:** tuberculosis, Rasch analysis, psychometric properties, perception, attitude assessment

## Abstract

**Introduction:**

Tuberculosis (TB)-related knowledge is an important evaluation metric for health education interventions. Factor analysis is limited when used on ordinal scales and does not provide in-depth item function examinations, whereas Rasch analysis addresses these limitations and offers potential advantages such as generalizability, testing of unidimensionality, producing an ordered set of items, and identifying poorly functioning items. Therefore, this research aims to develop a reliable and valid measure of perception and attitude toward TB (PATT) for public application use Rasch Analysis.

**Methods:**

A questionnaire-based survey was conducted on the Indonesian general population using the Google Form platform. Rasch analysis was then employed to examine the psychometric properties and develop the final items of PATT.

**Results:**

Experts from across the TB community participated in the PATT development, producing an initial scale of 16 items. Up to 1,616 participants completed the PATT questionnaire, where 74.8% were female, and 5% had a TB history. The final unidimensional 16-item scale has an item reliability of 1.00 for the two components (perception and attitude), a person reliability index of 0.87 and 0.60, as well as a Cronbach’s test reliability of 0.88 and 0.88 for perception and attitude, respectively.

**Conclusion:**

The PATT is a unidimensional scale with good construct validity and internal consistency. It has the potential to be useful for the assessment of TB perception and attitude in research and clinical practice.

## 1. Introduction

Tuberculosis (TB) is a communicable disease serving as a major initiator of other diseases and one of the top causes of mortality worldwide. The World Health Organization (WHO) estimated that the number of deaths officially related to TB in the world in 2020 (1.3 million) was nearly twice that of HIV/AIDS (0.68 million), and TB mortality has been more severely influenced by the COVID-19 pandemic in 2020 than HIV/AIDS (1, 2). It affects the lungs and can potentially spread to other body organs (3). *Mycobacterium tuberculosis* (MTB) is a bacteria that is transmitted through droplets and close contact with pulmonary TB patients, so it is important to stop the spread by treating patients and controlling the disease through case identification (4).

Currently, the gold standard for parameters of effective therapy is a focus on improving microbiological outcomes (5, 6). Moreover, public health also plays a crucial role in the therapeutic success of TB patients by addressing the physical, mental, and social pain that they experience (7). It becomes important because some studies mentioned that people with TB and their families may face bias and unfavorable attitudes like blame, humiliation, and a sense of being judged (8). It is also in line with other studies which found a link between perception, lack of awareness, and unfavorable social attitudes toward TB (9, 10). The result showed that patients hide their illnesses, disregard their therapy, and withdraw from society. Based on these facts, it is essential for health educators to accurately assess public understanding and then deliver education where the need exists, improving the rollout of public awareness and health promotion to increase perception and attitudes toward TB.

Recently, the development of valid and reliable tools to measure Perception and Attitude Toward Tuberculosis (PATT) in general populations is still scarcely investigated, with previous research focusing on students (11) and patients (12). Factor analysis is the most frequently used method to assess structural validity (13). However, there are known limitations to the use of factor analysis on ordinal scales, including its parametric basis and the emergence of ‘difficulty factors’, which may spuriously indicate multidimensionality (14). Furthermore, factor analysis does not provide in-depth examinations of item function in terms of targeting, differential item functioning, and local item dependencies, whereas Rasch includes such assessments (15, 16). Rasch analysis allows for generalizability across samples and items, considers that response options may not be psychologically equally spaced, allows for testing of unidimensionality, produces an ordered set of items, and identifies poorly functioning items as unexpected responses. Each of these characteristics becomes a potential advantage to be exploited (15). Besides, Rasch analysis has been performed for psychometric evaluations to assess the perception or attitude of dengue infection (17), stress scale (18), persistent shoulder pain (19), cancer (20), and the Covid-19 vaccine (21). However, no questionnaire comprehensively measures the perceptions and attitudes toward TB in the general population using Rasch analysis. The objective of the current study is to develop and validate the TB questionnaire to measure the perceptions and attitudes toward TB in the general Indonesian population on a large population from 34 provinces using Rasch analysis.

## 2. Materials and methods

### 2.1. Population and sample

The research population consisted of communities from the 34 provinces in Indonesia. The inclusion criteria were as follows: Indonesian citizens who are aged 15–64 years, can use and understand the Google Form platform, can read well, and are willing to fill out informed consent for approval. The participation in this study was completely voluntary. The convenient sampling was used to obtain a diverse pool of participants and snowball sampling, as participants were also encouraged to share the questionnaire within their personal networks. A sample size of 250–500 is sufficient for Rasch analysis and can produce good output for estimating item locations (16, 22).

### 2.2. Data collection

Data were collected using an online questionnaire for 4 weeks from May 30 to June 27 2022. Relying on a network of communities in various provinces in Indonesia, the Google form Survey link was distributed to the general community using social media platform “WhatsApp” and snowball sampling approach within a network of communities (23). Respondents’ information from google form was then extracted, coded, and entered into SPSS version 26.0 (IBM Corp, IBM Software Business Analytics) and exported into Winsteps software version 5.2.1.0.

### 2.3. Ethical approval and consent to participate

Approval was received from the Health Research Ethics Committee of Universitas Harapan Bangsa, Indonesia (B. LPPM-UHB/956/05/2022) in May 2022. All participants were informed about this research and signed digital informed consent form before participants could proceed to answer the questionnaire.

### 2.4. Questionnaire design

#### 2.4.1. Literature review and item generation

The initial step in developing the instrument was to identify the most representative variables in three domains (Table 1). These were identified and selected based on a literature review of studies conducted across globe including Malaysia (24), Pakistan (25), Buthan (26), and government guidelines of the Republic of Indonesia regarding the National Strategy for Combating Tuberculosis in Indonesia 2020–2024 (27). Based on these variables, a draft of the instrument for the general population Assessment Tools for Perception and Attitude of Tuberculosis (PATT) consisting of 16 questionnaire items was produced. Perception refers to a person’s understanding or awareness of tuberculosis (TB), which revealed the extent to which people stigmatize TB and TB sufferers, which is an important factor that can negatively impact the control and prevention of the disease. The concept of attitude is defined as a person’s overall emotional response, beliefs, and opinions regarding TB, including their views on who is responsible for addressing the problem, the importance of educating the community about TB, the adherence to TB treatment, and their attitudes toward stigmatizing and discriminating against TB patients.

**Table 1:** PATT questionnaires contents.

Domain	No. of item	Measurements	Response choices
Demography	3	Age, sex, history of TB	Closed-ended questions
Perception	10	Community perception on self-comfort and self protection toward TB sufferers	1 = Strongly disagree, 2 = Disagree, 3 = Not sure, 4 = Agree, 5 = Strongly agree
Attitude	6	Community attitudes toward TB sufferers, trusted sources of information, government policies, and medication for TB	1 = Strongly disagree, 2 = Disagree, 3 = Not sure, 4 = Agree, 5 = Strongly agree

#### 2.4.2. Face and content validity

Face validity is used to evaluate whether an indicator appears to be a reasonable measure in terms of word order, structure, order, and assessment format (23). The validity of the draft instrument’s contents (16 PATT items) was evaluated using experts in the field consist of 3 pharmacists, 1 specialist, and 2 public health professionals who have experienced in the treatment of TB (28, 29). The same expert was then sent with a fresh copy, a quick 4-point Likert scale, a cover letter detailing the research goals, the content specifications for the research instrument validation, and comprehensive descriptions for evaluating the items. Their suggestions and observations led to the implementation of these adjustments. They were asked to individually express their views on the Likert scale regarding the tool’s applicability, clarity, completeness, and simplicity (29, 30).

The expert opinions about the content validity of the scales are quantified using the content validity index technique (CVI) (28, 29). An item is considered relevant if its content validity index (I-CVI) is higher than 0.79. It needs to be adjusted if it is between 0.70 and 0.79. It is judged irrelevant if it is lower than 0.77 (29). Similar to this, the CVI scale (S-CVI) was estimated using the expert (S-CVI/UA) and CVI mean (S-CVI/Ave) techniques (29). S-CVI/UA values between 0.80 and 0.90 have excellent content validity (28, 29). Our results used six experts, and the acceptable CVI values were at least 0.83 (31).

#### 2.4.3. Psychometric analysis

This section was based on the Research Population and Sampling Techniques for the Rasch Measurement Model Construct validity and reliability. Respondents that participated in the validation of the 16-item PATT instrument were selected from the population, comprising subgroups of active people in the 34 provinces of Indonesia. This approach was recommended to obtain the greatest number of respondents to calculate the ideal number of estimations.

#### 2.4.4. Reliability test

Internal consistency and reliability are used to determine the extent to which the items in each of the 16 PATT items subscales measure the same concept. A person or item reliability of ⩾0.6 (32) and a person or item separation index of ⩾1.5 indicate good internal consistency and reliability (16). A Cronbach’s alpha test reliability of a minimum of 0.6 also supports good internal consistency and reliability (32).

#### 2.4.5. Validity test

Fit statistics analysis determines the extent to which the data fit the Rasch model for both items and persons and the whole subscale. These statistics are based on the mean square (MNSQ) and Z-standardized scores (ZSTD). The ideal MNSQ outfit and infit values are 1 based on the Rasch measurement model. However, values ranging from 0.5–1.5 are still acceptable (15). The point measure correlation range of 0.4–0.85 can also be seen as an additional indicator (15, 33), together with infit and outfit ZSTD values between −2 and +2 (34). The outfit is the outlier-sensitive fit statistic, which is based on the conventional chi-square statistic. This is more sensitive to people’s unexpected observations on items they find relatively easy or difficult, and vice versa. Infit (inlier-pattern-sensitive fit statistic), known to be based on the chi-square statistic, with each observation weighted by its statistical information (model variance). This is more sensitive to unexpected patterns of observations by people on items aimed at them, and vice versa (16).

#### 2.4.6. Bubble chart

A person-fit analysis was applied to assess the reliability of the person-response relationship. Moreover, finding people with unusual reaction patterns can be useful. Item fit analysis was conducted in the meantime to determine whether PATT instrument items can accurately assess the perception and attitude of Indonesians toward TB sufferers. This can be achieved through the pattern of item distributions in terms of item difficulty and person ability using the item person map and bubble chart. Item person mapping is an important Rasch representation that depicts an illustration of a person’s ability and item difficulty of an underlying construct (15). A specific item should be improved or removed once it is a misfit. In this research, the item fit analysis was described with a bubble chart (16). The bubble chart demonstrated the item fit order, with the horizontal axis (X) signifying item fit statistics that showed the community has a higher ability than the item difficulty level. On the other hand, the vertical axis (Y) indicated the item measure (16). This graph compared item difficulty to mean-square (MNSQ) in- and out-fit. Every question in the questionnaire was represented by a circle, with each item’s size being according to the calibration standard error. Regardless of how the fit and item interactions as well as the fit and person interactions were plotted, things should ideally be close to a logit value of 1 for infit and outfit MNSQ (15). The bubble chart is also a Rasch representation capable of helping to determine items that fall within the expected range (15). The validity of a scale is threatened when there are more misfit (underfit) items on the right-hand side of the bubble chart than overfit items on the left-hand side (15, 16, 35).

#### 2.4.7. Wright map

The distribution of the question difficulty level is depicted with the Wright Map, and the test information function is used to identify the ideal degree of community proficiency for this instrument (36). The two domains of perception and attitude were examined. The Wright map illustrates the item-person interactions using the same linear scale (logit scale). This analysis aimed to determine how well item difficulty distribution corresponds to the general level of perception and attitudes among Indonesians. The difference between the participants’ ability and the items’ average (M) difficulty can be used to gauge the correspondence. Hence, a greater fit between both scores, with a difference of 0 indicates that the participant’s aptitude and the item’s difficulty are perfectly matched (15, 35). Provided the difference is greater than 1 logit, it indicates the difficulty of the questions or objects is more than what the community can handle (15, 16). The item-person research in the Wright map shows people’s abilities on the left and objects’ difficulty on the right, to better understand how items and people interact with one another. It is also possible to evaluate people and things’ manner of interactions with one another and look at how well-suited each person is to fit into society. The community has a 50% chance (*p* = 0.5) of providing the appropriate response once the item indication matches the person’s indicator, as seen in the Wright map. This is because the item’s difficulty is inversely correlated with the community’s aptitude. In case the individual’s indicator is higher than the item’s indication, the community has a greater than 50% chance (*p* > 0.5) of providing the correct response (16, 37). However, once the individual’s indicator is lower, the community’s chance of providing the correct response is less than 50% (*p* = 0.5) (15, 16).

## 3. Results

### 3.1. Demographic characteristic

**Figure 1:**
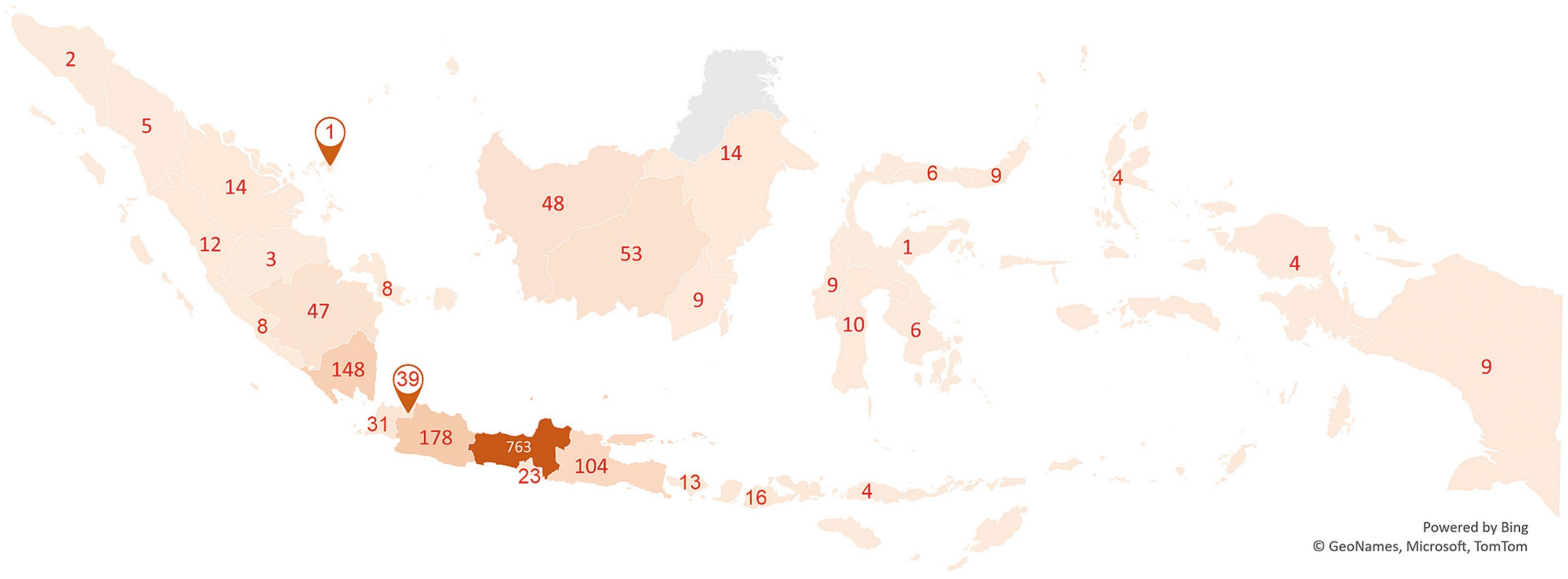
Respondents of PATT questionnaire.

A total of 1,616 PATT participants completed the questionnaire from 34 Indonesian provinces as indicated in see Figure 1. This is a larger sample than the minimum sufficient sample size required for Rasch analysis to produce good output for estimating item locations (16, 22). Our results show that almost three-fourths of the participants were women (74.8%), and most of the participants were aged 17–25 years (60.8%). Most of the respondents (95%) never had a history of TB, and 50.6% were from senior high school education level (Table 2).

**Table 2:** Demographic characteristics of the participants (*N* = 1,616).

Baseline characteristics	*n*/*N* (%)
Sex	
Female	1,208/1,616 (74.8%)
Male	408/1,616 (25.2%)
Age	
17–25 years	982/1,616 (60.7%)
26–35 years	430/1,616 (26.6%)
36–45 years	150/1,616 (9.3%)
46–55 years	47/1,616 (2.9%)
56–65 years	6/1,616 (0.4%)
>65 years	1/1,616 (0.1%)
History of tuberculosis	
Yes	81/1,616 (5.0%)
No	1535/1616 (95.0%)
Education level	
Elementary School	12/1,616 (0.7%)
Junior High School	21/1,616 (1.3%)
Senior High School	817/1,616 (50.6%)
Bachelor Degree	580/1,616 (35.9%)
Master Degree	175/1,616 (10.8%)
PhD Degree	11/1,616 (0.7%)

### 3.2. Face and content validity

Face validity is a kind of content validity that considers how clear, appropriate, and relevant the questions of a certain instrument are ‘on its face’ when respondents complete them (29). It should be noted that only pertinent variables ought to be collected and employed for a draft instrument design. Each component that a medical practitioner added to the instrument while it was disassembled was employed precisely when combined. None of the curator’s items was found to have a significant issue with structure or phrasing, even though numerous adjustments were made based on suggestions and guidance from other experts to strengthen the instrument’s face validity. Lynn (31) acknowledged that 3- or 5-point rating scales might be considered, but also advocated using a 4-point scale to avoid having a neutral and ambivalent midpoint. In this research, labels for the four points were 1 = Not relevant at all, 2 = Item needs some revision, 3 = Relevant but needs minor revision, and 4 = Very relevant. Using the Lynn descriptor. Using the Lynn descriptor (31), the validity check results for 16 items were obtained, and each item had a CVI (I-CVI) score of at least 0.80 for relevance, clarity, and completeness (Table 3). Average CVI (S-CVI) outcomes in expert (S-CVI/UA) with a mean CVI (S-CVI/Ave) approach are in the range of 0.88 and 0.98 for relevance, simplicity, and completeness on a scale. Therefore, each of the 16 questions on the draft PATT questionnaire should be considered to improve public perception and attitude toward TB.

**Table 3:** Content validity of PATT instruments, perception (10 items), and attitude (6 items).

Variable Construct	Panel 1		Panel 2		Panel 3		Panel 4		Panel 5		Panel 6		Expert in Agreements	I-CVI Score	S-CVI Score
	*n*	Code	*n*	Code	*n*	Code	*n*	Code	*n*	Code	*n*	Code			
Perception															
P1	4	1	4	1	4	1	3	1	4	1	4	1	6	1.00	1
P2	4	1	4	1	4	1	4	1	3	1	4	1	6	1.00	1
P3	3	1	3	1	4	1	4	1	4	1	4	1	6	1.00	1
P4	4	1	4	1	4	1	3	1	4	1	4	1	6	1.00	1
P5	4	1	4	1	4	1	3	1	4	1	4	1	6	1.00	1
P6	4	1	4	1	4	1	4	1	4	1	4	1	6	1.00	1
P7	4	1	4	1	4	1	4	1	4	1	4	1	6	1.00	1
P8	4	0	4	1	4	1	3	1	4	1	4	1	5	0.83	0
P9	4	1	4	1	4	1	4	1	4	1	4	1	6	1.00	1
P10	4	1	4	1	4	1	4	1	4	1	4	1	6	1.00	1
Attitude															
A1	4	1	3	1	4	1	4	1	4	1	4	1	6	1.00	1
A2	4	1	4	1	4	1	4	1	4	1	4	1	6	1.00	1
A3	4	1	4	1	4	1	4	1	4	1	4	1	6	1.00	1
A4	3	1	4	1	4	1	4	1	4	1	4	1	6	1.00	1
A5	4	1	4	1	3	1	4	1	4	1	4	1	6	1.00	1
A6	4	1	4	1	2	0	3	1	4	1	4	1	5	0.83	0
Mean		0.938		1.000		0.938		1.000		1.000		1.000	Summary	15.67	14.00
The average proportion of items judged as relevant across the 6 experts												0.979	Average	0.98	0.88

I-CVI, Item Content Validity Index; S-CVI/UA, Scale Content Validity Index; n, score of items relevance; P, perception; A, Attitude.

### 3.3. Psychometric analysis

#### 3.3.1. Reliability test

Table 4 summarizes the item reliability and separation index for the logit scale produced by the RASCH analysis. The item reliability for the two components (perception and attitude) is all 1.00, with a high separation index over the minimum permitted value of >1.5 (16). This indicates 16 items in the PATT questionnaire have good item reliability index values based on reliability thresholds of at least 0.6, which is also reflected in the separation index score for each construction (16, 32).

**Table 4:** Reliability and separation index of PATT instruments, perception (10 items), and attitude (6 items).

Variable constructs	ID item	Item measure		Person measure		Cronbach’s alpha
		Reliability	Separation	Reliability	Separation	
Perception	P1 – P10	1.00	27.24	0.87	2.63	0.88
Attitude	A1 – A6	1.00	39.63	0.61	1.23	0.68

The person reliability measures for perception and attitude were 0.87 and 0.60, respectively, which all fell within the acceptable range of ≥0.6 (32). The results of Cronbach’s alpha reliability test were positive and accepted at a minimum value of 0.6, meaning the correlations for each of the remaining questionnaire items for perception and attitude were 0.88 and 0.68, respectively (32).

#### 3.3.2. Validity test

One item in perception (item P2) was detected to have a model misfit and highlighted with numbers in bold on the instrument, leaving a total of 15 items (9 items for perception and 6 items for attitude) toward TB (Table 5), based on recommendations from Bond and Fox (15) for the range of fit index values required to ensure acceptance. The results of the rerun Rasch analysis showed that the mean squared values (MNSQ) of 9 items of the perception that make up the logit scale were between 0.73 and 1.39 for the MNSQ infit and 0.81 to 1.38 for the MNSQ outfit, while the corresponding standard mean values (ZSTD) are between −3.63 and 5.13 for the infit ZSTD and − 3.69 to 7.64 for the outfit ZSTD (15, 16). Similarly, all of the items with PTMEA Corr polarities were positive, and most had high correlations with the constructs they were tested against, but few of them deviated from the suggested ranges of 0.19 and 0.78 [Table 4; (15, 16)]. However, the results showed some item/person misfits based on the ZSTD threshold (*n* = 16). Since the sample size was 1,616 participants (more than 200), the ZSTD requirement might be neglected (34).

**Table 5:** Items fit and misfit indices of PATT.

Item	Description	Infit		Outfit		PTMEA
		MNSQ	ZSTD	MNSQ	ZSTD	EXP.
Perception						
P1	You feel uncomfortable around people with TB	1.0867	2.4511	1.1772	4.3712	0.6252
P2	You think TB sufferers have the same social problems	1.8298	9.9018	2.0519	9.9021	0.4416
P3	You think TB sufferers are disgusting	0.9184	−2.3591	0.9074	−2.2891	0.6998
P4	You think the family of TB sufferers should not participate in any function	1.0082	0.2410	0.9827	−0.389	0.6779
P5	You think TB sufferers are scary	0.8786	−3.6791	0.862	−3.8991	0.7298
P6	You will keep your distance if you meet with TB sufferers	0.937	−1.8691	0.944	−1.5291	0.6454
P7	You feel afraid of TB sufferers	0.6582	−9.8993	0.6527	−9.8993	0.7616
P8	You try to have no contact with TB sufferers	0.9462	−1.6091	0.9304	−2.0091	0.6846
P9	You do not want to communicate with TB sufferers	0.8583	−4.2791	0.8684	−3.5391	0.7106
P10	You prefer to exclude TB sufferers from your community	0.8289	−5.3192	0.8002	−5.9292	0.7381
Attitude						
A1	TB is a serious and deadly disease	1.2532	5.8713	1.2753	4.3213	0.3159
A2	TB is a mutual problem between communities and the government	1.2582	7.0413	1.111	2.7611	0.7274
A3	TB education is needed in the community	0.5742	−9.8994	0.3668	−9.8996	0.7853
A4	The community should recognize the correct and appropriate TB information	0.8662	−2.6791	0.4584	−7.3895	0.6867
A5	TB treatment can be discontinued if the patient feels better	0.9680	−0.8390	1.3148	7.5713	0.1926
A6	Avoiding TB sufferers is the best attitude to prevent transmission	0.5742	−9.8994	0.3668	−9.8996	0.7853

#### 3.3.3. Bubble chart

**Figure 2:**
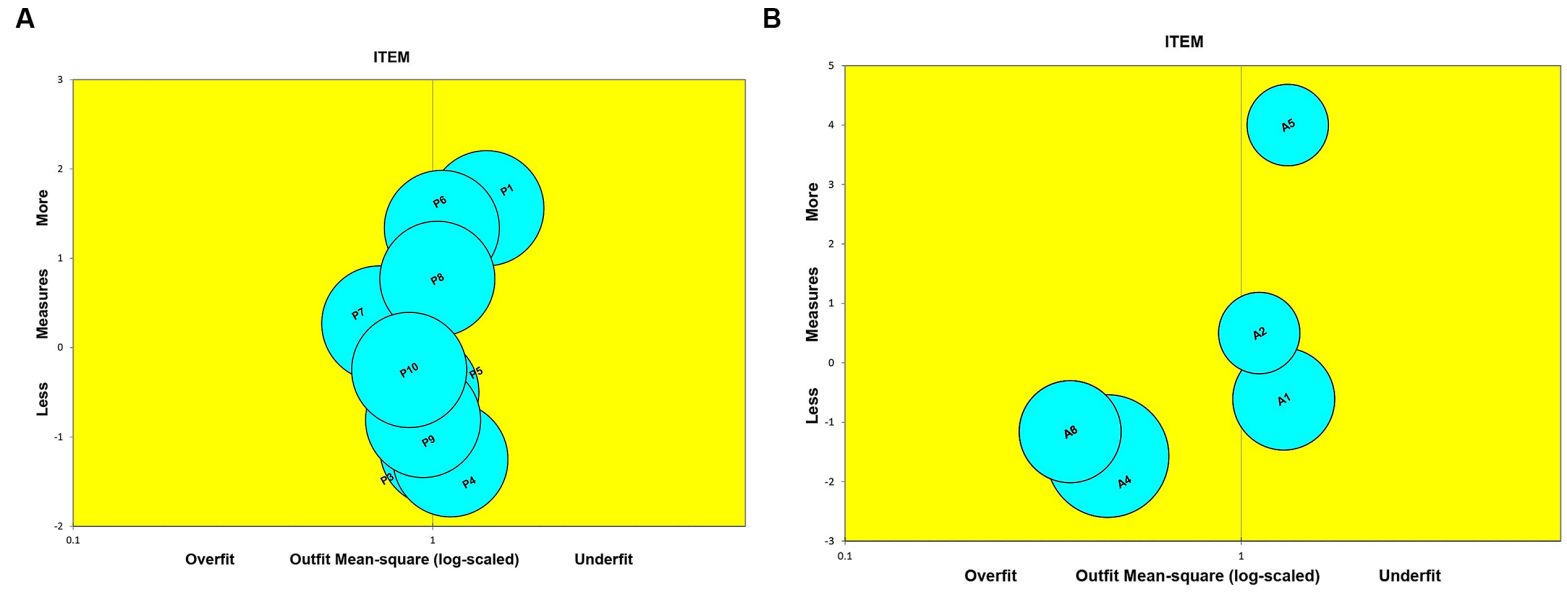
Bubble Chart **(A)** Perception and **(B)** Attitude toward TB explained the item fit order based on infit MNSQ; Y axis is JMLE measure; X axis is item fit mean square; overfit (*x* > 1.50); outfit (0.50 < *x* < 1.50).

As indicated in the bubble chart presented in Figure 2, no items are placed outside of the expected range (+2 and −2) and close to the Outfit Mean-square (log-scaled) =1. The circles and squares’ size indicates the measures’ precision (standard errors) along the vertical axis, i.e., the latent variable (15, 16). The horizontal axis is the fit, with overfit on the left (the responses are too predictable) and underfit on the right (the responses are too unpredictable), while the fit indicates the measurements accuracy. The P4 and A4 items were the easiest to sign, but the P1 and A5 were the hardest to sign.

#### 3.3.4. Wright map

**Figure 3:**
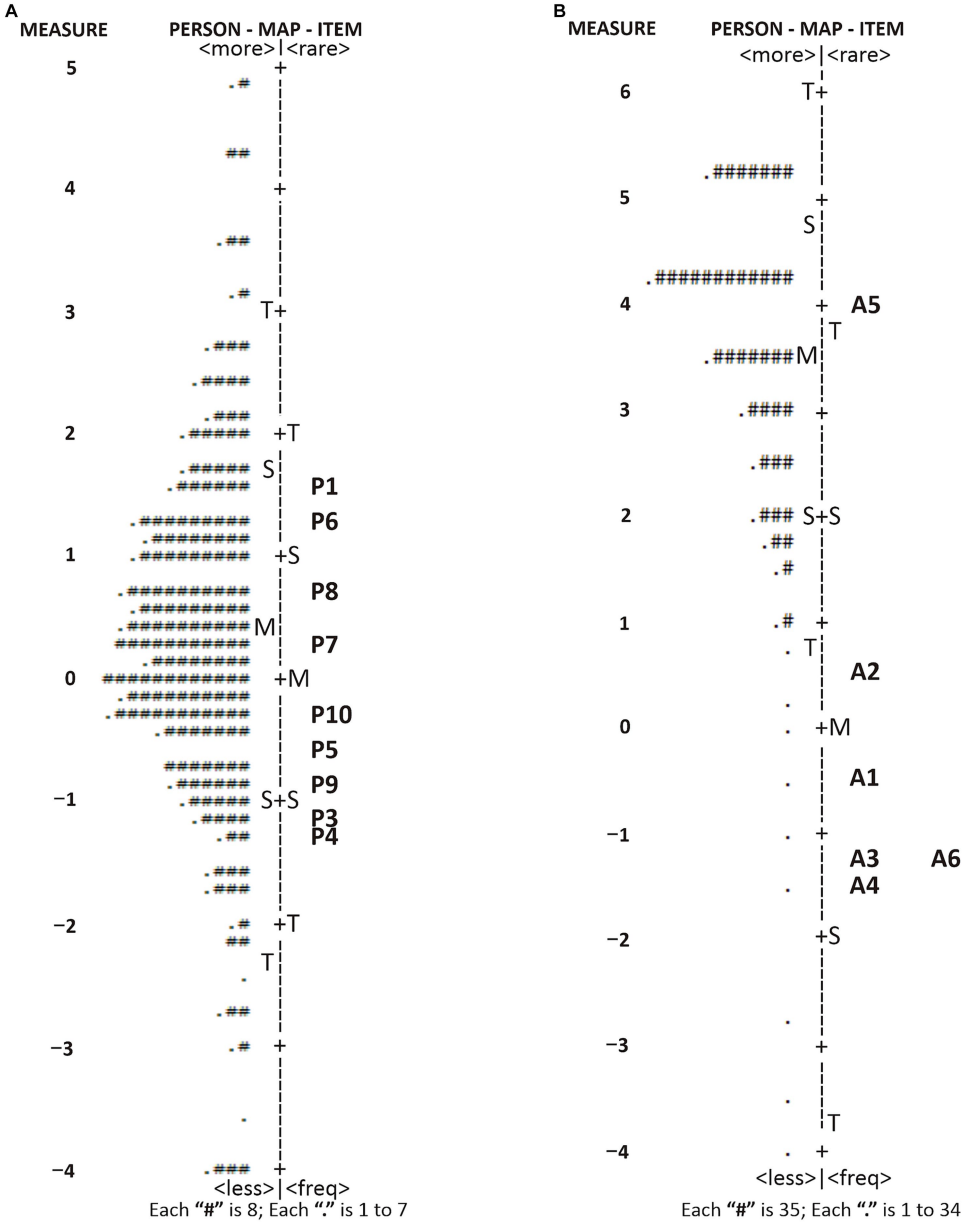
Wright Map of PATT instruments representing a direct comparison of person dispersion and item distribution. **(A)** perception and **(B)** attitude toward TB.

The perception domain in the PATT questionnaire shows the least frequent perception was P1, and the most frequent was P4 (Figure 3A). The attitude domain shows the least approved item was A5, and the most approved was number A4 (Figure 3B). Therefore, it can be highlighted that the test developed matches the target subgroup in terms of examining the perception and attitude toward TB in the 34 provinces’ communities.

## 4. Discussion

This research offers information on the general population’s PATT. Furthermore, it is the first to investigate the psychometric properties of the Indonesian-PATT using Rasch analysis, which was more appropriate for examining cross-cultural equivalency for psychiatric measures with a tendency of being non-normal (38). The Rasch analysis of the Indonesian-PATT indicated that the developed instrument was reliable, and inferences made from the item and person measures plus the fit information were valid reflections of the population’s perception and attitude.

Previous research assessed PATT using a descriptive cross-sectional method and found that although the level of knowledge and attitude toward TB among patients is satisfactory, the perception of this infectious disease is poor (39). TB diagnosis can create self-stigma, which tends to affect treatment success, because of the fear and perception of being isolated and discriminated (40). Therefore, stigma-reduction strategies need to be part of the national response to TB.

Overall, the PATT has acceptable psychometric properties related to accuracy, precision in measurement, and applicability. PATT is unidimensional, indicating that it only measures TB-related perception and attitude. Its item reliability was very high (reliability index of 1.00), well above the minimum threshold of 0.60 (32). This was higher than the Clinician Support for Patient Activation Measure belief and attitude assessment (41) as well as other infection knowledge tests (COVID-19) (42). It suggests the items on the PATT scale can locate a latent measure with a high level of precision, and good internal consistency and reliability.

In the context of the PATT, the personal characteristic called ‘ability’ may be conceived as one’s individual perception and attitude about TB; in other words, a person with higher scores on the PATT will have a higher level of agreement with individual perception and attitude. Moreover, the item characteristic of ‘item difficulty’ in the Rasch analysis represents in this context the propensity of an item to obtain systematically high or low scores when measuring the latent trait of interest. The item difficulty thus reflects the level of relevance each item in the tool has for the measured aspect.

Furthermore, one item in perception (item P2) was deleted, leaving 15 items because of the model misfit detected. Social issues are the type of question unfavorable to the general population. It contradicts a report by Ali (43), which examines the impact of social and economic factors on individuals and recommends taking them into account when treating TB. A combination of unfavorable social factors (low income, hard physical labor, smoking, alcohol abuse, malnutrition, low hygienic level), as well as a separate factor (income per family member), have the most significant influence on TB morbidity (44). Based on this fact, differentiation evidence of social pressure in individuals with TB might be related to improving TB outcomes.

This research is valuable due to being the first to evaluate the psychometric qualities of the Indonesian-PATT using Rasch analysis. The PATT instrument enables the development of public perception and attitude profiles. The objective is that healthcare professionals and policymakers would be able to deliver systematic and thorough education about issues requiring resolution in the community. Additionally, the PATT is expected to serve as a guide for the government while developing more specialized TB control measures.

We realize that our instrument used was developed on 1,616 participants’ perceptions and attitudes toward TB. Consequently, the findings of our study could not be generalized to general settings in the 34 provinces in Indonesia because most respondents in our study were from the Java Island compared to respondents from other islands. Future research is required on a more diverse population and a balanced distribution of respondents between provinces in Indonesia. Another limitation is that Indonesia is a large country with cultural, historical, and social factors that could influence people’s perceptions and attitudes toward TB. Moreover, the utilization of instruments and development is projected to be applied in further studies with larger sample sizes and different locations.

## 5. Conclusion

This research indicated that the 16-items PATT developed is valid and reliable for measuring TB perception and attitude among the general population in Indonesia. The instrument can be used to evaluate the public perception and attitude profiles concerning TB. The goal is for policymakers and healthcare professionals to provide systematic and in-depth education on community problems that need to be solved. Additionally, the PATT is expected to serve as a guide for the government while developing more specialized TB control measures in the community.

## Data availability statement

The raw data supporting the conclusions of this article will be made available by the authors, without undue reservation.

## Ethics statement

Approval was received from the Health Research Ethics Committee of Universitas Harapan Bangsa, Indonesia (B. LPPM-UHB/956/05/2022) in May 2022. All participants were informed about this research and signed informed consent.

## Author contributions

All authors listed have made a substantial, direct, and intellectual contribution to the work and approved it for publication.

## Funding

This study was funded by Directorate of Research and Community Service Universitas Padjadjaran, grant numbers 1549/UN6.3.1/PT.00/2023 and supported by the Center of Excellence in Higher Education for Pharmaceutical Care Innovation Universitas Padjadjaran.

## Conflict of interest

The authors declare that the research was conducted in the absence of any commercial or financial relationships that could be construed as a potential conflict of interest.

The reviewers SF and WR declared a shared affiliation with the authors DP and IP to the handling editor at the time of review.

## Publisher’s note

All claims expressed in this article are solely those of the authors and do not necessarily represent those of their affiliated organizations, or those of the publisher, the editors and the reviewers. Any product that may be evaluated in this article, or claim that may be made by its manufacturer, is not guaranteed or endorsed by the publisher.
